# Social Robots for Evaluating Attention State in Older Adults

**DOI:** 10.3390/s21217142

**Published:** 2021-10-28

**Authors:** Yi-Chen Chen, Su-Ling Yeh, Tsung-Ren Huang, Yu-Ling Chang, Joshua O. S. Goh, Li-Chen Fu

**Affiliations:** 1 Department of Psychology, College of Science, National Taiwan University, Taipei 10617, Taiwan; yijhen0924@gmail.com (Y.-C.C.); trhuang@ntu.edu.tw (T.-R.H.); ychang@ntu.edu.tw (Y.-L.C.); joshuagoh@ntu.edu.tw (J.O.S.G.); 2 Center for Artificial Intelligence and Advanced Robotics, National Taiwan University, Taipei 10617, Taiwan; 3 Neurobiology and Cognitive Science Center, National Taiwan University, Taipei 10617, Taiwan; 4 Graduate Institute of Brain and Mind Sciences, College of Medicine, National Taiwan University, Taipei 10051, Taiwan; 5 Department of Neurology, National Taiwan University Hospital, College of Medicine, National Taiwan University, Taipei 10048, Taiwan; 6 Department of Computer Science and Information Engineering, National Taiwan University, Taipei 10617, Taiwan; lichen@ntu.edu.tw; 7 Department of Electrical Engineering, National Taiwan University, Taipei 10617, Taiwan; 8 MOST Joint Research Center for AI Technology and All Vista Healthcare, Taipei 10617, Taiwan

**Keywords:** aging, social companion robot, sustained attention, mind-wandering, cognitive evaluation

## Abstract

Sustained attention is essential for older adults to maintain an active lifestyle, and the deficiency of this function is often associated with health-related risks such as falling and frailty. The present study examined whether the well-established age-effect on reducing mind-wandering, the drift to internal thoughts that are seen to be detrimental to attentional control, could be replicated by using a robotic experimenter for older adults who are not as familiar with online technologies. A total of 28 younger and 22 older adults performed a Sustained Attention to Response Task (SART) by answering thought probes regarding their attention states and providing confidence ratings for their own task performances. The indices from the modified SART suggested a well-documented conservative response strategy endorsed by older adults, which were represented by slower responses and increased omission errors. Moreover, the slower responses and increased omissions were found to be associated with less self-reported mind-wandering, thus showing consistency with their higher subjective ratings of attentional control. Overall, this study demonstrates the potential of constructing age-related cognitive profiles with attention evaluation instruction based on a social companion robot for older adults at home.

## 1. Introduction

With advanced science and technology, the longevity revolution is taking place worldwide. By 2050, one in six people will be over the age of 65 [1]. As older adults are the main growing demographic group, technological solutions are urgently required to meet the constantly increasing demands of care services. In this regard, active aging—the lifestyle that maintains positive subjective well-being by having good physical, social, and mental health in old age [2]—is becoming a trend in the era of population aging. Specifically, active aging is supported by one’s ability to sustain attention for goal-oriented behaviors. However, there are great variations in sustained attention among older adults; deficiency in sustained attention, on the other hand, is often associated with detrimental performances in everyday tasks such as paying bills or driving [3,4]. Early identification of subtle impairments in attention is therefore critical to planning treatment, and so are intervention strategies before the consequences become detrimental.

Early disease detection for older adults has also been envisioned as an appealing option for effective in-home care [5]. For example, algorithms using data from the hospital and an experiment on predicting regional chronic disease of cerebral infarction have reached 94.8% accuracy [6]. Although such a detection rate seems optimistic, a significant obstacle often lies with the data gaining stage, during which sophisticated equipment is needed for collecting required neurological data. Under these circumstances, participants are usually tested in venues, such as hospitals or research facilities. However, several reasons could impede older adults from receiving such assessments in a place outside of their home environment. One of which is the challenge of traveling between homes and research facilities for individuals with disabilities, a common problem in aging populations. In the United States, two in five adults aged 65 years old or above have at least a disability in hearing, vision, mobility, cognition, self-care, or independent living [7]. Moreover, most older adults with mild cognitive impairments may lack awareness of their condition, which often prevents them from accessing appropriate health care [8,9]. Furthermore, the drastic changes that have been brought by the worldwide pandemic over the past two years, coronavirus disease (COVID-19), have been declared a global public health emergency. Governments worldwide have implemented lockdown policies that limit activities or access to resources and facilities to contain the spread of the virus. This unprecedented pandemic has forced people to embrace a home-bounded lifestyle and re-envision the possibilities for many in-home care facilities.

Concerning these issues, services provided by robotic technology have recently added new perspectives that focus on using social companion robots as a medium for collecting data on psychological evaluations in home-like environments. Social robots are distinct from non-robotic digital services by having physical features that enable users to interact with machines in a manner more closely resembling interactions with humans [10]. For older adults, assessments mediated by robots with sociability are more enjoyable and accessible than other remote assessment methods [11]. Growing evidence suggests that it is feasible to implement cognitive assessments (traditionally conducted by human experimenters) using social robots and to collect data via human-robot interaction (HRI) by targeting older adults and those with mild cognitive impairment [12] for robot-administered screening for global cognitive functions [13,14].

The tests mentioned above are standard screening measures in clinical and research settings that often involve data gathered through verbal communication. However, autonomous operation for fluent verbal communication is still a substantial challenge in robotics, and many socially assistive robots still adopt teleoperated control methods [15]. Yet, for older adults, verbal capacity is deemed the most practical yet least reliable feature on a socially assistive robot [16]. Hence, the technical limitations of verbal communication in robots could reduce the ecological validity of robot-mediated assessments in older adults [17]. Additionally, a recent study has developed a framework in which social robots could conduct regular clinical screening interviews in geriatric care. The study suggested that it is essential to incorporate multimedia touch screen systems, besides the robot itself, to enable interactions with visual stimuli [18]. This is especially important for older adults with difficulties in technological proficiency. Thus, a touch device that incorporates more intuitive interactive features is essential to facilitate cognitive assessments with HRI approaches. In order to increase the accessibility for home-based robot-mediated cognitive assessments, here we propose a promising alternative to capture the subtle but noteworthy cognitive and functional changes in aging with a behavioral paradigm presented by a touch screen tablet with a social robot experimenter.

Evidence from clinical neuropsychology has shown the potential of using sustained attentional tasks to detect subtle impairments in attentional control [19] and of rehabilitating neurological conditions [20]. Moreover, the individuals’ sensory-motor responses assessed through such behavioral paradigms were sensitive to various age-related risks, including falling and physical frailty [21], hearing loss [22], and metabolic risks [23]. All these findings suggest that the behavioral paradigm test for attentional control is a good candidate for developing an age-related health risk detection system in the home environment. Furthermore, robot performances should be less susceptible to verbal-related mistakes since the behavioral features during attentional control are accessed using a standardized task format with brief and easy instructions.

Sustained attention is defined as the ability to maintain endogenous attention over an extended period [24], and it can be assessed using the Sustained Attention to Response Task (SART, [25]). In the SART, a variant of GO/NO-GO tasks, participants are instructed to respond to the non-targets by pressing a key (the GO trials) and to withhold their response to the targets (the NO-GO trials). With a higher proportion of GO trials and a relatively low proportion of NO-GO trials, automatic responses to GO trials are supposed to be dominated during the task. Failing to withhold a response to the NO-GO trials (i.e., the targets), referring to an error of commission (EoC), is indicative to a transient attentional lapse. Importantly, older adults tend to show longer reaction times (RTs) preceding EoC, whereas longer RTs are associated with reduced EoC in older adults’ performances [26,27,28,29]. By contrast, omission errors, referring to the failure to respond to the GO non-targets, are greater in older adults than younger adults [30]. In terms of the subjective aspect of attentional states, studies have shown that older adults are more capable of inhibiting *mind-wandering* (MW, referring to the phenomenon of attentional shift from the current task to task-unrelated thoughts) than younger adults [30,31,32,33]. In contrast, compared to younger adults, older adults tend to report more task-related thoughts, which involve thoughts about one’s performance of the ongoing task [27,30,32].

The trend of longer RTs with fewer EoC in older adults has suggested an age-related speed-accuracy trade-off in the SART. This effect has also been evident in other studies [34,35,36,37] and has been interpreted as an endorsement of a more conservative strategy to maintain attentional control as people age [38]. The increased EoC is also reflective of an automatic state of behavioral control associated with an increased frequency of task-unrelated MW (e.g., [39,40,41]). Consistently, the less frequent MW in older adults could partially explain the minimal age differences in the sensitivity of identifying the NO-GO targets in the SART [42], indicating the reduction of MW as a compensatory strategy in older adults. In parallel, more frequent task-related thoughts in older adults also suggest that they deploy more time in the attentional state towards the external task and, thus, are less consciously aware of their internal state [41]. According to Cheyne et al. [43], this reflective state for past performances can be observed by increased omission errors. In older adults, increased omissions have been found to be particularly prominent in those who reported more task-related thoughts [30].

Task difficulty has been recognized as a critical factor in modulating the degree of EoC and omissions. Specifically, as task difficulty increases, the leverage between the two types of errors (i.e., EoC and omissions) tend to skew to the salient perceptual cues, such that individuals deliberatively slow their responses in preparation for targets, thus reducing the EoC, but inevitably producing more omissions [44]. These results align with the evidence that older adults tend to engage a greater top-down cognitive control to perform at the same proactive error (i.e., EoC) rate as younger individuals [34,36]. While performing a GO/NO-GO task, an increased activation level in the anterior cingulate cortex was found in older adults, relative to younger adults [45], suggesting higher cognitive control involvement for older adults. In summary, evidence from the SART indicates a stronger engagement of top-down cognitive control in older adults that contributes to specific consequences of having better response selection (i.e., fewer NO-GO errors) and fewer self-generated distractions (i.e., MW). Yet, there is a greater chance of missing the timing of action (i.e., more omissions) as well as engaging more performance reflection (i.e., task-related thoughts).

It is well established that there is a degradation in multisensory integration, response inhibition, and selective attention [46,47,48,49] as a consequence of aging. As a result, it can be challenging for older adults to maintain daily functioning (e.g., navigating safely and driving). A compensatory mechanism featuring a conservative response pattern is indicated to contribute to a relatively preserved sustained attention in older adults in response to these age-related cognitive changes. Therefore, the SART, and its consistent age-related pattern, could be applied in identifying individuals with sustained attentional failures and early detection of various age-related health risks. Specifically, individual performances outside the expected norm for their age group could be a potential marker for age-related risk of cognitive decline [24]. Moreover, the SART has been widely applied and studied with a website version [38], making it an ideal candidate for evaluating the feasibility of in-home cognitive assessments of older adults.

Therefore, in this present study, we examine whether the well-established age effect on sustained attention can be observed via a social robot cognitive assessment framework in a home-like environment. We expected to see that, compared to younger adults, healthy older adults would exhibit (1) lower NO-GO error rates (EoC) and increased omission errors. Also, a slower and more cautious response pattern was anticipated for older adults, which is characterized by (2) a longer RT and a lower response tendency towards the GO stimuli. In addition, we introduced two probe questions during the SART to examine the subjective evaluation of attentional states. For these attentional probes, we anticipated (3) an age-related reduction of self-reported MW in older adults. We also hypothesized (4) self-rated performances would be higher in older adults, who enact more top-down control to sustain attention. Finally, to replicate the findings that suggest the two error types correspond to different subjective attentive states [43], we expected to observe (5) a positive correlation between EoC and MW, as well as between omission and task-related thoughts.

## 2. Materials and Methods

### 2.1. Participants

The older adults’ ages ranged from 60–80 years old, and the younger adults’ ages ranged from 18–30 years old. There were 22 older adults (11 females; age = 71.74 ± 4.28) and 28 younger adults (14 females; age = 21.8 ± 2.36). There was no significant group difference in the educational level (years) of the two groups, suggesting a matched level of education, t(40) = 1.12, *p* = 0.27 (old = 13.8 ± 4.54; young = 14.96 ± 1.43), who enrolled in the study and provided signed informed consent for participating in the experiment. All participants had normal or corrected to normal visual acuity. None of them had any history of neurological or psychiatric disorders or any severe hearing or visual impairments. They were all recruited from local communities with a Mini-Mental State Examination (MMSE) score ≥ 24. This study was approved by the Research Ethics Committee at National Taiwan University (NTU REC: 201803HS017) and was implemented accordingly.

### 2.2. Social Robot

We used a programmable humanoid robot RoBoHoN (Sharp Co., Ltd. Osaka, Japan), a hybrid of robot and mobile phone telephony. It is a small (with a height of 19.5 cm while standing) and easily portable robot-shaped smartphone, with all the basic mobile phone functions, such as phone, camera, and a range of services accessed through Apps. RoBoHoN uses the Android 5.0 operating system, which is accessed using the 2-inch touchscreen on the back. It has built-in speech-to-text and text-to-speech engines that support speech recognition and production. To accurately detect sentence endpoints in participants’ speech and to manage relevant conversational contingencies such as speech pauses, repetitions, and queries that could arise during the task session, the RoBoHoN was controlled by a human operator. Unknown to the participants, the human operator was seated in an observation room and could over-write irrelevant verbal responses from the RoBoHon due to the inaccurate speech recognition. The RoBoHon was introduced as a playmate and guided the participants to finish a series of interactive tasks with verbal instructions (see ‘procedures’ for details). Although RoBoHon has a touch screen, it is located on the robot’s back and is too small to present stimuli. Hence, in the SART session, a tablet computer was implemented for task presentation. The RoBoHoN was placed on a table with a standing posture beside the tablet computer, and both devices faced the seated participant.

### 2.3. Modified Sustained Attention to Response Task

**Figure 1:**
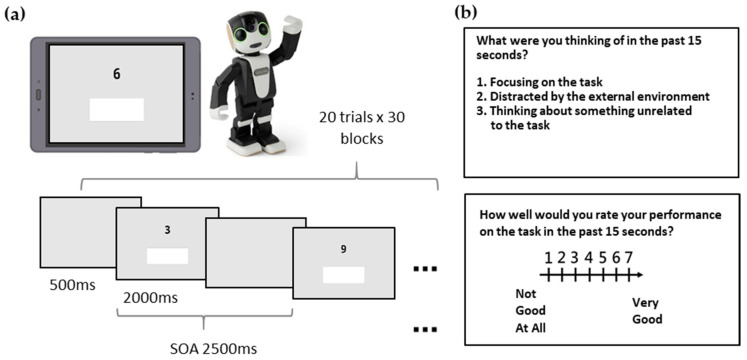
The paradigm of the modified SART. The example image in (**a**) shows the task presented on a tablet beside a RoBoHon robot. The participants were instructed to press the white response button as soon as possible each time a Go-stimulus was presented, if what was presented was a NO-GO target, the participants were required to withhold the button pressing response. (**b**) Illustrates the two probe questions that appeared at the end of each error block whenever any response error had been detected.

The Sustained Attention to Response Task (SART) was programmed with jsPsych on a web interface and integrated into an Android native App with Apache Cordova installed on an Asus ZenPad 3S 10 (Z500M). During the experiment, the ZenPad was placed on the table facing the participants’ eyes (with 25 cm distance), and no restrictions were made on the participants’ movements. The participants tapped the surface to perform the task (including pressing the response button and selecting probe questions). A digit selected from 0–9 was presented at the center of the screen for 2000 ms in each trial. Participants were instructed to press a button underneath the stimulus as soon as they saw a GO stimulus (digits from “0–9” except the digit “3”). If the stimulus was a NO-GO target (digit “3”), participants were required to withhold his or her response until the digit disappeared; the trial ended once the button was pressed or no response was given after 2000 ms (Figure 1a). A blank screen would appear between each trial for 500 ms. There were 18 GO trials and 2 NO-GO target trials (10%) presented randomly within each block and 30 blocks in total (20 trials per block).

If any error was detected within the 20 trials of a given block, the block would be defined as an error block and would trigger the following two probe questions (Figure 1b). The first probe question instructed participants to self-report their thoughts during the past 15 s. We used 15 s but not the whole block because there was no particular structure of blocks from the participants’ perspective, and a period of 15 s was within the range that participants could estimate their own state of attention and respond truthfully from a pilot study. Participants were instructed to choose the closest statement among the following options: “Focusing on the task, which includes the task-related thoughts about the performances”, “Distracted by the external environment”, or “Thinking about something unrelated to the task”. Options displayed on the screen include a statement about on-task, external distraction, or task-unrelated MW. The second probe question was, “How well would you rate your performance on the task in the past 15 s?”, and this question was rated on a 7-point scale, where a higher score indicated a better self-reported performance.

The self-aware attentiveness rate was calculated by dividing the occurrence of each attentional state by the number of error blocks. Likewise, the mean self-reported performance was calculated by dividing rating scores by the number of error blocks. If no errors were detected within the given block, there would be no probe questions at the end of the block, and the next block would begin seamlessly. This latent block design kept participants from becoming familiar with the task structure, allowing their attention to fluctuate naturally without being interrupted by preparing for a sure-to-come NO-GO target.

### 2.4. Questionnaires

Two neuropsychological questionnaires (Chinese version) were implemented, the Pittsburgh Sleep Quality Index (PSQI) and the Mindful Attention Awareness Scale (MAAS). The PSQI, embedded with 19 self-report items that included seven components (subjective sleep quality, sleep duration, sleep latency, sleep disturbance, sleep efficiency, daytime sleepiness, and medication use), was adopted to measure the quality and patterns of sleep [50]. The MAAS, a 6-point Likert scale with 15 items, was used to measure participants’ trait of mindfulness [51]. It took around 20 to 30 min to complete both questionnaires.

### 2.5. Procedure

The study was conducted in an observation room equipped with furniture that resembles a residential environment. Participants were told that a human experimenter would stay at the corner of the room, but she would not intervene in the human-robot interaction unless any technical problems occurred with the robot. After receiving the written consent form from the participants, a humanoid robot, RoBoHoN, stood on the table and began to introduce itself as the opening of this experiment. This experiment constituted of a series of tasks, including the robot-administered cognitive testing, robot-accompanied toy-playing, and the modified SART, with several brief human-robot conversations interspersed in between. The whole experiment was bundled with the three tasks in order to mimic a scenario of using social robots in a home-like environment. The cognitive testing included assessments with cognitive functioning in verbal fluency, episodic memory, prospective memory, and aspects of executive function. The toy-playing session was designed for the RoBoHoN to learn the toy preference of each participant, who were each given one minute to play with each of our three toys, before a five-minute free play with all the three toys. The data obtained from the cognitive testing were analyzed and reported elsewhere [12]. Those data were not included in the current study.

In the SART session, the RoBoHoN verbally instructed the participants to direct their attention to the experimental pad on the desk, where the instruction of the SART was presented. After confirming that participants had no further questions about the instructions and completed the SART practice session with 20 trials, the participants were instructed to rate their current sleepiness level (pre-task sleepiness) ranging from 0–4 before the formal session of the SART. After the participants finished 15 blocks, they could decide to rest for 5 to 10 min or go straight to finish the rest 15 blocks. Overall, it took approximately 30 min to finish the SART and 1.5–2 h to finish the whole experiment, including the other sessions.

### 2.6. Statistical Analyses

Data were analyzed using SPSS version 20 (IBM Corp., 2013). We investigated four objective attentional indexes collected during SART, which were (1) EoC, the ratio of failing to withhold a keypress response when presented with the NO-GO target (digit “3”) to the total block number, (2) omission, the ratio of missing a keypress when presented with GO stimuli to the total block number, and (3) mean RTs of responding correctly to GO stimuli.

Correspondingly, by taking a signal detection approach with the hit rate ($P_{Hit}$: correctly withhold the response to NO-GO target), false alarm rate ($P_{FA}$: missing response to GO stimuli), and correct rejection rate ($P_{CR}$: correctly respond to GO stimuli), we calculated response bias (*β*: the inclination of responding to GO stimuli under uncertainty) with the following equation:

(1) $$\beta =\frac{f_{SN}\left(c\right)}{f_{N}\left(c\right)}=\frac{f\left(z_{Hit}\right)}{f\left(z_{CR}\right)}$$

In the signal detection theory [52], both the signal and the noise distributions can be estimated based on the standard deviation (i.e., the z-score) of the probabilities associated with each distribution. Individuals make their decision relative to the threshold c, where a signal will be reported as present when the internal signal is above c and absent when the internal signal is below c. The z-value associated with the probability of a hit ($P_{Hit}$) will reflect where c is positioned relative to the signal distribution ($f_{SN}$). Similarly, the z-value associated with the probability of a *CR* ($P_{CR}$) will reflect the position of c relative to the noise distribution ($f_{N}$). Response bias (β) can be calculated as the ratio of the height of $f_{SN}$ to $f_{N}$ at the given threshold c. By assuming that both the $f_{SN}$ and the $f_{N}$ follow a Gaussian distribution (f(x) with mean = 0 and standard deviation = 1), the bias β can be computed by the ratio of the function values of $z_{Hit}$ to $z_{CR}$. The$\;z_{Hit}$ and $z_{CR}$ are calculated by the z-transformed value of $P_{Hit}$ and $P_{CR}$, respectively.

For extreme cases, such as $P_{Hit}$ = 100% or $P_{FA}$ = 0%, the standard procedures proposed by Snodgrass and Corwin [53] were applied with this equation:

(2) $$\begin{array}{c} {\hat{P}}_{Hit}=\frac{y_{Hit}+0.5}{N_{NG}+1}\\ {\hat{P}}_{FA}=\frac{y_{FA}+0.5}{N_{G}+1} \end{array}$$

It is not possible for humans to make no mistake, and the extreme values for $P_{Hit}$ or $P_{FA}$. are caused by the limited number of trials. In the cases of extreme values, ${\hat{P}}_{Hit}$ and ${\hat{P}}_{FA}$ would be applied to estimate the hit and FA under sufficient trials. In Equation (2), the $y_{Hit}$ and $y_{FA}$ represent the number of trials that were classified as hit and false alarm, and $N_{NG}$ and $N_{G}$ denote the number of NO-GO and GO trials.

In addition, two subjective ratings, including the (1) self-aware attentiveness (on-task, distracted, or MW) and (2) self-performance assessed during the end of each error block, were also compared across the two age groups.

Task outliers were identified if the data lay three standard deviations (SD) away from the group mean within each group. According to this criterion, three older adults (EoC outliers) and five younger adults (one omission outlier, two EoC outliers, and two RT outliers) were excluded from further analyses. Subsequently, 19 older adults (11 females; age = 71.89 ± 4.46) and 23 younger adults (14 females; age = 21 ± 1.31) were included in further data analyses. The demographic information is shown in Table 1.

We examined the age effect in sustained attention performance by controlling potential confounding factors with one-way analyses of covariance, ANCOVAs, with the α level of 0.05. The controlled variables were (1) MAAS, which is inversely associated with MW propensity during sustained attention [31]; (2) scales related to daytime sleepiness, the PSQI, which correlates with reduced attentional control [54]; and (3) the sleepiness-before-task, as the control variables to eliminate possible confounds in the age effect. Additionally, we calculated Pearson correlation coefficients between the attentional indices and self-rated evaluations (attentiveness and performance) to validate the relationships between the objective measures and subjective attentional control ratings.

## 3. Results

### 3.1. SART Performance

**Figure 2:**
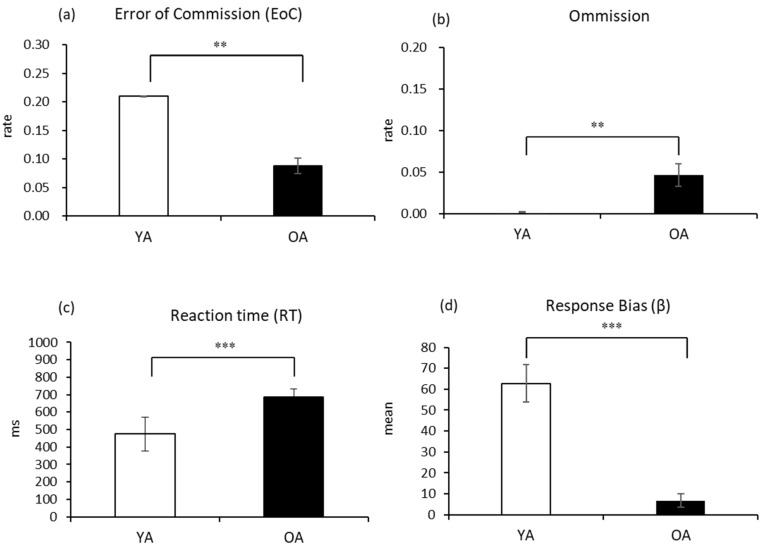
Age differences in types of errors committed and response patterns. (**a**) The propensity to commit EoC was higher for younger adults, while (**b**) omission was higher for older adults. (**c**) Mean RT was higher for older adults. (**d**) Response bias was greater for younger adults, indicating a stronger inclination in responding to GO targets. Error bars represent one standard error from the mean. YA: younger adults; OA: older adults; ** *p* < 0.01, *** *p* < 0.001.

There was a significant age effect on EoC, omission, RT, and β (Table 2). Compared to younger adults, older adults had fewer EoCs, more omission errors, longer RTs, and lower βs (Figure 2). That is, when seeing a NO-GO target (the target “3”), older participants exhibited a stronger tendency, compared to their younger counterparts, to withhold pressing keys (no-response), which helped them make fewer commission errors and yet more omission failures when seeing a GO stimulus. The longer response latency and lower response bias also support the conclusion that older adults tend to engage in a slow and cautious response strategy (i.e., a more conservative response tendency) to avoid inhibition failures.

### 3.2. Self-Reported Attentional Control

**Figure 3:**
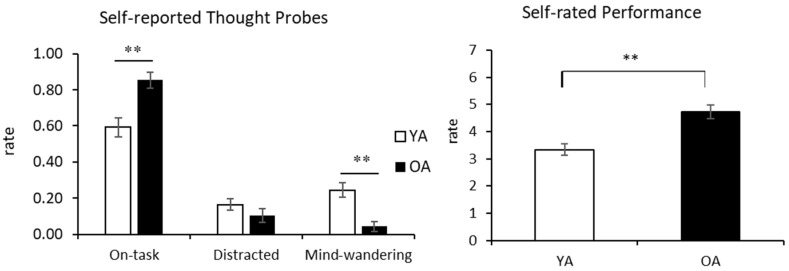
Age differences in self-aware attentiveness and performance. Results are shown separately for self-reported thought probes and performance. The frequency of reporting on-task and being MW significantly differed among age groups. In terms of self-rated performance, older adults tended to rate higher about their performance. Error bars represent one standard error from the mean. ** *p* < 0.01.

The results of one-way ANCOVAs demonstrated that age had an effect on both on-task and MW, but not on distracted attentional state (Table 3). Older adults reported more focused attention than younger adults and rated themselves as less MW. They also rated themselves with higher scores in the self-rated performance (Figure 3). That is, older adults reported more on-task thoughts and less MW than younger adults, which was consistent with their higher self-rated attentional control.

### 3.3. Relationships between Objective and Subjective Performances of Sustained Attention

Pearson product-moment correlation coefficients were computed to assess the relationships between objective and subjective indices of SART performance, combining the data of older adults and younger adults. As shown in Table 4, a greater EoC rate was associated with a lower self-rated performance, r = −0.49, *p* = 0.001, and more omission errors were correlated with more frequently reported on-task thoughts, r = 0.32, *p* = 0.04, less MW rate, r = −0.31, *p* = 0.04, and higher self-rated performance, r = 0.40, *p* = 0.008. In parallel, RTs showed a similar pattern with subjective indices: longer RTs were correlated with more frequently reported on-task thoughts, r = 0.30, *p* = 0.05, lower MW, r = −0.37, *p* = 0.016, and higher self-rated performance, r = 0.51, *p* = 0.001. Furthermore, a lower response bias to GO targets was associated with a better self-rated performance, r = −0.39, *p* = 0.01.

## 4. Discussion

### 4.1. Strategic Shift in Sustained Attention by Older Adults

The present study demonstrated the effect that age has on sustained attention through assessment with the modified SART. Critically, this effect could be replicated using a tablet with the HRI approach in a home-like environment. Our main results revealed a well-documented, age-related strategic shift. Consistent with the existing literature, older adults tended to adopt a conservative strategy and thus showed fewer EoC. Most have considered that this speed-accuracy trade-off in older adults is tactically driven [26,45,55]. The adoption of a speed-accuracy trade-off in SART performance is associated with various aspects of functioning among community-dwelling seniors. Specifically, longer RTs might reflect the greater engagement of compensatory mechanisms to maintain the accuracy level for the NO-GO target (i.e., reduced EoC). Presumably, older individuals who suffer from more executive declines exhibit greater reliance on this compensatory strategy than those who do not. This view is evident by findings showing that older adults who evince longer RTs in sustained attentional tasks are more likely to have a history of falling [21], which is usually accompanied by impaired executive functioning [47].

Notably, unlike EoC that signifies inhibition failures in older adults when adopting a more cautious strategy, increased omission errors were found despite more self-aware attentional resources being applied in the task. This phenomenon could be explained by the association between omissions and the task-driven attentional state—the task-related thoughts [43], which are not often consciously made aware by the participants [41]. Our finding supports this explanation by showing increased omissions associated with more task-relevant thoughts and higher self-reported performances, suggesting such errors were made unconsciously and therefore were excluded from self-rated capacity under attentional control. Interestingly, aging is not always associated with more omission errors. For example, according to Zavagnin et al. [30], among older individuals, only those with an average age of 80 years old (age range: 75–85) produce significantly more omissions than younger adults, but not those with an average age of 69 years (age range of 65–74). Specifically, age was only associated with increased omissions when targets were presented under high uncertainty (i.e., noisy conditions, [56]). Therefore, a possible explanation is that the design of the latent blocks (i.e., no apparent block structure for the participants) in our modified SART reduced the possibility for participants to predict and prepare for the NO-GO target in each block. Compared to the traditional paradigm, our study’s increased temporal uncertainty can be considered as a more difficult condition and is used to avoid the ceiling effect in most studies (e.g., [31,57]). Hence, it seems that only when the task becomes demanding do the task-related thoughts result in observable age-related differences in omission errors.

Aligned with the current evidence, a decrease in overall MW and increased on-task states were reported by older adults. Critically, patients with Alzheimer’s disease (AD) at early stages have been observed to have reduced MW during the SART [58]. Nonetheless, by taking into account the SART performances, the study revealed that patients with AD were more dependent upon the speed-accuracy trade-off than healthy older adults. These findings indicated that solely the self-reported thought probes are not adequate enough to reflect the actual subjective experiences in individuals with early-stage cognitive impairments. Hence, in addition to the increased MW, the behavioral pattern that includes increased errors and RT should also be considered when profiling an individuals’ attentional state. It is worth noting that, in our study, only omission errors were associated with reduced MW, with no association between EoC and MW. Building on the conjecture mentioned earlier, this could be due to this study’s more difficult task demand, which prompted participants to rely heavily on the strategy of slowing down to avoid inhibition errors induced by MW. This implies that the strategic shift is more relevant to reducing MW than inhibiting habituated responses, per se.

### 4.2. Evaluation of Sustained Attention in Older Adults

To our knowledge, this is the first study that demonstrated the well-reported age effect on sustained attentional capacity using the HRI approach. In the scope of using robotic technology for early cognitive impairments detection, many studies have focused on implementing clinical screening assessments with HRI. However, most have assessed only specific aspects of attention, such as processing speed, selectivity, and capacity of transient attention [14,59]. Among these, attentional selectivity and capacity—referring to the ability to focus on a specific source of stimuli while ignoring others and the amount of information encoded simultaneously, respectively—both showed linear declines with age [60]. On the contrary, sustained attention is distinctive from other aspects of attention by showing only minor age-related decrement [26,61]. Notably, once an impaired sustained attention signal appears, even with just a minor sign (i.e., faster RT) among community-dwelling seniors, it is sufficient enough to detect individuals at risk for various health issues, including falling [21,62], hearing loss [22], and metabolic risks [23]. These facts highlight that sustained attention should also be utilized in the early detection system provided by home-based ambient assistance.

### 4.3. Social Robots for Older Adults’ Cognitive Evaluation

The results in this present study offer a possibility that older adults can perform this psychological paradigm autonomously with a social robot’s accompaniment. Under the HRI context, older adults in this study showed a typical conservative strategy that is analogous to the results observed with computer-based lab tasks. Accordingly, introducing social robots as experimenters can elicit decision-making processing in participants that resemble the processes as guided by human experimenters. Specifically, natural vocal intonation constitutes an important factor for humans to apply in social norms when engaging with a robot [63]. In our study, the social robot exhibited its sociability to participants before testing sessions with verbal interactions, suggesting that it is critical for inducing social presence. Social presence, the perception of co-presence with another social agent, is the essence of social robots. Social presence has been implicated in various aspects of interpersonal interaction and can increase the intention to use a robot [64], enhance psychological consolations [65], facilitate rapport building [66], and guide human behavior efficiently [67]. Despite having these advantages of HRI, many improvements still need to be made before introducing social robots into the healthcare context, including voice recognition and accent interpretation [68], which have been rated the least reliable of features on social robots [16].

### 4.4. Using the Tablet as an Extension Device

In the present study, we demonstrated an alternative to utilizing the advantage of social robots and accurately assessing older adult cognitive functions simultaneously. After the greeting sessions, the RoBoHon guided participants to the instructions presented on the tablet, and the tablet itself also recorded the task responses. In addition, the practice trials before the formal session were also able to help identify any misunderstanding regarding the task instructions and minimize task confounding due to the robot’s verbal ability. Compared to traditional computers, the tablet has a more intuitive graphical interface that is more accessible to users and has been widely accepted by older adults, who might be novice tablet users [69]. Indeed, 41 % of 65- to 69-year-olds reported having tablet computers [70], making the tablet an ideal digital device to pair with a social robot. The expanding usage of social robots could be a solution to the increasing need for healthcare services. To this end, robots should elicit social presence and aim to be lightweight and low-cost [71]. Our study indicates that the RoBoHon, a cell-phone-based robot with built-in natural language ability, or any similar sized social robot (e.g., Zenbo, Jr.) paired with tablet computers, can serve as a remote cognitive assessment set up for older adults. This setting can be applied to a wide range of visually presented cognitive tasks; for instance, performing a longitudinal assessment on visual memory tasks that are sensitive to the gradual decline in subjectively reported mild cognitive impairment (e.g., CANTAB, [72]).

### 4.5. Future Directions

A major restriction for using the tablet as an extension device is the limited data storage capacity. Hence, we were unable to record fine-grained data of sustained attention in this study. For instance, in healthy adults, a brain activity-based MW detection system has identified specific saccade patterns during the SART and could also be used as an indicator of attentional states [73]. Such a limitation, nevertheless, can be overcome as technology continues to advance. For example, the recent technological release for portable eye-tracking devices has the potential to help cognitive screening assessments detect transient attentional states. In addition, the restorative effects of aerobic exercise on cognitive loss [74] can also be utilized in residential environments with the extension of social robots. By adding wearable sensors on foot, individuals are able to exercise with the cognitive simulative protocol [75]. In these settings, not only the elderly but the individuals with impaired motor abilities (e.g., injuries by accident or illness) who need to engage in rehabilitation programs persistently can benefit from the social robots. Overall, under the increasing demand for social robots, the cost-efficient approach of pairing social robots with commercialized biometric sensing tools could be an agonist used to popularize social robots in healthcare scenarios.

Another issue that had not been addressed in this study was how individual differences in digital proficiency and acceptance affect the incentives of using social robots. Older adults represent a highly heterogeneous group of technology users, and those with lower socioeconomic backgrounds tend to have lower Internet skills [76]. Additionally, the skill set and intention for using new technology varied among older adults, with some who reported using digital services only because there was no offline alternative, whereas others tended to use them more but needed someone to help them try out digital services that were unfamiliar or difficult for them to use [77]. All these suggest a potential barrier for introducing social robots to those older adults who have lower digital awareness. Nonetheless, technology acceptance could be enhanced by well-designed digital services. For instance, older adults showed a higher level of engagement in interacting with an AI-based coaching application than younger adults, despite that it requires a certain level of digital literacy (e.g., navigate the in-app menu, pair connected Bluetooth devices with their mobile phones) [78], suggesting that older adults, in general, would engage with new technologies when provided the opportunity. Future studies are needed in terms of how the underlying factors of digital awareness interact with the intentions and the further experiences of using social robots by older adults.

Social robots are not merely tools for humans. Unlike other digital tools, robots with various degrees of sociability would affect individuals’ acceptance of robots [79,80,81]. Robots with exquisite social abilities, such as those alleged to have advanced feelings like pain and fear, are sometimes troubling to adult users [82]. In the meantime, social robots are expected to have multiple roles for their multi-functionalities despite interacting with the same individual [83]. For instance, the social robot in our study, RoBoHon, is expected to be applied under various HRI scenarios, including providing emotional comfort for children [84] and eliciting memory retrieval for seniors [85] with its wide range of social abilities (e.g., chatting, joking, and dancing). Therefore, future research is warranted regarding how different social features affect the impressions and expectations toward a robot with various roles.

The tide for deploying social robots in residential environments is on the rise. We are in an urge to address the overwhelming needs of healthcare that come with an aging society. The homebound lifestyles amid COVID-19 also facilitate a transformation of in-person healthcare. By having social robots at home, the aging population can remain in their familiar environment and sustain their self-sufficient active lifestyle while also maintaining a higher level of communication and monitoring of their health. In addition to intelligently interacting with the environment, the techniques developed must be successfully adopted by users. Therefore, it is essential to consider the cost-efficient aspect for potential users. This study illustrates that a cognitive behavior paradigm that presents visual stimuli with a tablet is compatible with a potential robot-assistive cognitive evaluating system, and the results obtained are consistent with the burgeoning existing literature. Nevertheless, future research is needed for more versatile applications that will be required given the trend of an aging society.

## 5. Conclusions

In this study, we introduced a robotic experimenter under a home-like setting for a sustained attentional task. Using the tablet computer presented stimuli and recorded task responses; the significant results of this study dovetail with major consensus discovered by standard psychometric paradigms—sustained attention is preserved by the recruitment of a conservative response strategy in older adults. Specifically, the indices associated with a conservative response strategy, represented by longer RTs and increased omissions, were reflective of the subjective attentional states and could be utilized to identify the engagement of such compensatory effects in older adults. Failing to deploy this strategy would indicate non-normative performance for a person in this given age group and would link to an increased risk of falling in older adults, and it also serves as an early signal of cognitive impairment. Furthermore, the attentional profile demonstrated in this study provides a basis for identifying an individual’s sustained attentional states from younger to older age, which is particularly essential in this era of distractions.

## Figures and Tables

**Table 1:** Demographic information of participants.

	Older Adults			Younger Adults		
	Range	Mean	SD	Range	Mean	SD
Age (years old)	65–80	71.89	4.46	19–24	21.00	1.31
Education (years)	1–21	13.84	4.54	12–17	14.96	1.43
MMAS	46–87	66.53	10.23	41–85	59.61	10.04
PSQI	1–14	4.89	2.71	4–13	6.96	2.62
Pre-task sleepiness	0–1	0.32	0.48	0–2	0.83	0.65

Note. SD standard deviation; MAAS: Mindful Attention Awareness Scale; PSQI: Pittsburgh Sleep Quality Index.

**Table 2:** The age effect on SART performances.

SART Indices	Younger Adults Mean (SD)	Older Adults Mean (SD)	Statistics
EoC (rate)	0.21 (0.12)	0.09 (0.09)	F(1,37) = 10.06, *p* = 0.003, *η*p² = 0.21
Omission (rate)	0.002 (0.003)	0.05 (0.06)	F(1,37) = 12.27, *p* = 0.003, *η*p² = 0.25
RT (ms)	473.99 (49.20)	685.51 (97.42)	F(1,37) = 51.41, *p* < 0.00, *η*p² = 0.58
β	62.79 (42.40)	6.76 (14.10)	F(1,37) = 19.51, *p* < 0.00, *η*p² = 0.35

Note. SD: standard deviation.

**Table 3:** The age effect on self-reported attentional control.

Thought Probes	Younger Adults Mean (SD)	Older Adults Mean (SD)	Statistics
On-task (rate)	0.59 (0.26)	0.85 (0.20)	F(1,37) = 9.57, *p* = 0.004, *η*p² = 0.21
Distracted (rate)	0.16 (0.16)	0.10 (0.17)	F(1,37) = 0.79, *p* = 0.38, *η*p² = 0.02
MW (rate)	0.24 (0.19)	0.04 (0.12)	F(1,37) = 12.56, *p* = 0.001, *η*p² = 0.25
Self-rated performance	3.34 (1.0)	4.73 (1.13)	F(1,37) = 10.81, *p* = 0.002, *η*p² = 0.23

Note. SD standard deviation.

**Table 4:** Bivariate correlation between SART performances and subjective ratings of attentiveness.

	On-Task	Distracted	Mind-Wandering	Self-Rated Performance
EoC	0.01	−0.13	0.10	−0.49 **
Omission	0.32 *	−0.15	−0.31 *	0.40 **
RT	0.30 *	−0.06	−0.37 *	0.51 **
β	−0.11	−0.03	0.18	−0.39 *

Note. * *p* < 0.05, ** *p* < 0.01.

## Data Availability

The data supporting this study’s findings are available on request from the corresponding author, S.-L.Y. The data are not publicly available due to their containing information that could compromise the privacy of research participants.
